# Sperm Proteome after Interaction with Reproductive Fluids in Porcine: From the Ejaculation to the Fertilization Site

**DOI:** 10.3390/ijms21176060

**Published:** 2020-08-22

**Authors:** Chiara Luongo, Leopoldo González-Brusi, Paula Cots-Rodríguez, Mª José Izquierdo-Rico, Manuel Avilés, Francisco Alberto García-Vázquez

**Affiliations:** 1 Department of Physiology, Veterinary School, International Excellence Campus for Higher Education and Research (Campus Mare Nostrum), University of Murcia, 30100 Murcia, Spain; chiara.luongo@um.es; 2 Department of Cell Biology and Histology, Faculty of Medicine, University of Murcia, 30100 Murcia, Spain; leopoldo.gonzalez@um.es (L.G.-B.); paula.cotsr@um.es (P.C.-R.); mjoseir@um.es (M.J.I.-R.); 3 Institute for Biomedical Research of Murcia, IMIB-Arrixaca, 30100 Murcia, Spain

**Keywords:** pig, spermatozoa, biofluids, fertility, proteomics

## Abstract

Ejaculated sperm are exposed to different environments before encountering the oocyte. However, how the sperm proteome changes during this transit remains unsolved. This study aimed to identify proteomic changes in boar sperm after incubation with male (seminal plasma, SP) and/or female (uterine fluid, UF; and oviductal fluid, OF) reproductive fluids. The following experimental groups were analyzed: (1) SP: sperm + 20% SP; (2) UF: sperm + 20% UF; (3) OF: sperm + 20% OF; (4) SP + UF: sperm + 20% SP + 20% UF; and (5) SP+OF: sperm + 20% SP + 20% OF. The proteome analysis, performed by HPLC-MS/MS, allowed the identification of 265 proteins. A total of 69 proteins were detected in the UF, SP, and SP + UF groups, and 102 proteins in the OF, SP, and SP + OF groups. Our results showed a higher number of proteins when sperm were incubated with only one fluid than when they were co-incubated with two fluids. Additionally, the number of sperm-interacting proteins from the UF group was lower than the OF group. In conclusion, the interaction of sperm with reproductive fluids alters its proteome. The description of sperm-interacting proteins in porcine species after co-incubation with male and/or female reproductive fluids may be useful to understand sperm transport, selection, capacitation, or fertilization phenomena.

## 1. Introduction

In porcine species, billions of ejaculated spermatozoa are deposited within the female reproductive tract by natural or artificial insemination. In order to have successful fertilization, it is necessary that suitable spermatozoa reach the oviduct [1], after crossing the uterus and oviduct towards the oocyte and interacting with different environments [2,3]. Through these interactions, spermatozoa are lost and/or selected, with only a few of them able to reach the fertilization site [4,5,6,7,8,9]. The spermatozoa surface undergoes changes both in the male and female genital tracts [10,11], by absorbing components released to the fluids they encounter along the way [12,13]. During the ejaculation, spermatozoa are immersed in the first reproductive fluid, the seminal plasma (SP), a complex biological fluid containing inorganic ions, citric acid, organic salts, and proteins, which is produced by the testes, epididymis, and, mainly, accessory reproductive organs (vas deferens, seminal vesicles, prostate, and bulbourethral glands) [14]. The SP is involved in spermatozoa metabolism, survival, and transport within the female reproductive tract [15], which includes inhibiting the female immune response [16]. The uterus acts against spermatozoa by migrating polymorphonuclear (PMN) granulocytes [17,18] and this immune response may be modulated by SP, as it contains several cytokines interacting with the uterus epithelium, as previously shown in boar [19,20]. It has been shown in mice that SP contains SVS2 (seminal vesicle secretion 2), an SP protein involved in maintenance of the physiology of spermatozoa within the uterus [3]. Recently, we demonstrated that SP can protect spermatozoa against the hostile uterine environment by preserving sperm motility and acrosome integrity after 3 h of incubation [21]. Indeed, SP can be found within the female genital tract up to 4 h after insemination [22], and the presence of SP has been observed in the oviduct in mice [23]. Another characteristic is that the SP contains proteins that bind to the sperm membrane creating a protective layer to avoid damage during their passage within the uterus, with these proteins being involved in different processes, such as sperm survival and the final stages of sperm maturation [24,25]. Among the described boar SP proteins, the PSP-I/PSP-II spermadhesins are the most abundant. These proteins coat the spermatozoa surface and interact with the uterine immune response, declining the influx of PMN granulocytes [17,26]. Additionally, SP has decapacitating factors and proteins that stabilize the sperm surface in order to avoid early capacitation [27,28], such as spermadhesins AQN-3 and AWN-1 that bind to the sperm membrane, playing a decapacitation role by binding to the sperm surface until the capacitation occurs, and are also involved in zona pellucida binding [27].

Within the female reproductive tract, sperm flow through the uterine and oviductal environments, which themselves are key elements to the selection of the best spermatozoon for fertilization [29,30,31]. During the estrus cycle, changes in the proteome of the uterine and oviductal environment occur [32,33,34] and it is known that, within the uterus, some proteins, such as spermadhesin AWN, mucins, and complement cascade proteins, interact with gametes [32]. Once in the uterus, spermatozoa are in contact with the uterine fluid (UF), a fluid that contains ions, nutrients, hormones, growth factors, and proteins, as previously described in humans [29,35] and pigs [36,37]. After crossing the uterus, spermatozoa are ready to cross the utero-tubal junction and reach the oviduct, where they will be surrounded by oviductal fluid (OF). The OF is also a dynamic milieu that contain organic and inorganic molecules, originated from plasma transudation and glycoproteins secreted by oviductal cells [38], composing the environment in which fertilization takes place [32,39,40]. Among OF proteins, the most abundant are serum proteins, such as albumin and transferrin, that are involved in preventing oxidative stress [40]. It has been shown that incubation with OF induces functional changes in spermatozoa [41], and some of its proteins like OVGP1 could play a role in modulating spermatozoa function, improving viability, motility, and acrosome integrity [33,41,42]. Sperm functional changes can also be induced by hyaluronic acid, a molecule identified in both female and male reproductive fluids. Specifically, this molecule is found in exosomes adhered to the sperm surface following early capacitation and contributes to sperm survivability by interacting with the CD44 receptor present in the sperm plasma membrane [43]. Following these interactions with reproductive fluids, spermatozoa undergo different modifications, as described in other species [32,44,45]. Thus, the hypothesis of the study was that the interaction between boar spermatozoa and the different reproductive fluids could alter the sperm proteome. Through this proteomic analysis, it would be possible to enhance the knowledge regarding the effect of surrounding biological fluids on sperm. Therefore, the aim of the present study was to identify the proteins that adhere to the sperm surface after incubation with female reproductive fluids (UF or OF) in the presence or absence of SP.

## 2. Results

### 2.1. Proteins Identified in Different Biological Fluids

A total of 265 different proteins throughout the different fluids were identified. To minimize the risk of false positives, only proteins present in at least three out of the five replicates were considered, resulting in 122 final proteins. The list of identified proteins among the different incubation fluids, including the relative number of peptides of each protein, is shown in Supplementary Material File 1.

### 2.2. Identification of Sperm-Interacting Proteins According to Incubation with Different Reproductive Biological Fluids

#### 2.2.1. Sperm-Interacting Proteins after Incubation with UF and/or SP

**Figure 1:**
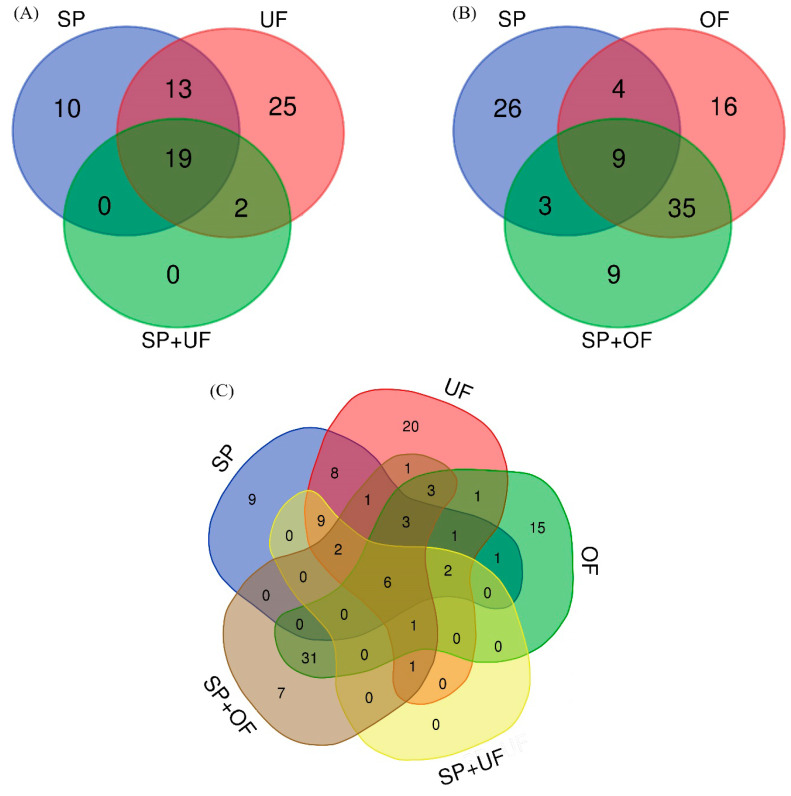
Venn diagram illustrating the number of different proteins in each incubation: the overlap of seminal plasma (SP), uterine fluid (UF), and seminal plasma plus uterine fluid (SP + UF) sperm-interacting proteins (**A**); the overlap of SP, oviductal fluid (OF), and seminal plasma plus oviductal fluid (SP + OF) sperm-interacting proteins (**B**); and the overlap of the different incubation fluids (**C**).

The number and the overlap of sperm-interacting proteins from SP, UF, and SP + UF are illustrated in a Venn diagram (Figure 1A). A total of 69 proteins were detected in spermatozoa incubated with SP, UF, and SP + UF. The exclusive proteins from each group and common proteins among the different experimental groups are shown in Table 1. Forty-two proteins were detected in spermatozoa incubated with SP, 10 of which were exclusive of this group. A total of 59 proteins were detected in spermatozoa incubated with UF (25 of them exclusive for this group) and 21 proteins in the SP + UF group. In the presence of SP + UF, the number of sperm-interacting proteins detected was lower than when spermatozoa were incubated with SP or with UF. Additionally, the comparison among SP, UF, and SP + UF identified 19 common proteins. Furthermore, 2 proteins were in common between UF and SP + UF: Apolipoprotein A-I and deleted in malignant brain tumors 1 protein (DMBT1). The comparison between the SP and UF groups identified 13 common proteins.

#### 2.2.2. Sperm-Interacting Proteins after Incubation with OF and/or SP

The number and the overlap of sperm-interacting proteins from SP, OF, and SP + OF are illustrated in a Venn diagram (Figure 1B). A total of 102 proteins were detected in spermatozoa incubated with SP, OF, and SP + OF. The exclusive proteins from each group and common proteins among the different experimental groups are shown in Table 2. Forty-two proteins were detected in spermatozoa incubated with SP, 26 of which were exclusive of this group. A total of 64 proteins were detected in spermatozoa incubated with OF and 56 proteins in the SP + OF group. Sixteen proteins were found exclusively in the OF group, and 9 proteins were exclusive of the SP + OF group. Additionally, the comparison among SP, OF, and SP + OF identified nine common proteins. Furthermore, 35 proteins were shared between the OF and SP + OF groups. The comparison between the SP and OF groups identified four common proteins.

#### 2.2.3. Sperm-Interacting Proteins after Incubation with Different Reproductive Fluids and Their Relationship

The number and the overlap of sperm-interacting proteins comparing identified proteins for the different incubation fluids and their relationship are illustrated in a Venn diagram (Figure 1C). Six proteins were in common among the SP, UF, OF, SP + UF, and SP + OF groups: Carbohydrate-binding protein AQN-1, fibronectin 1, seminal plasma sperm motility inhibitor, spermadhesin AWN, tubulin alpha chain, and tubulin beta chain. DMBT1 was shared between the UF, OF, SP + UF, and SP + OF groups, as well as apolipoprotein A-I with the exception of an absence in OF. Three proteins were in common among UF, OF, and SP + OF: Heat shock protein HSP 90-alpha, multifunctional fusion protein, and serum albumin.

### 2.3. Functional Analysis of Sperm-Interacting Proteins

**Figure 2:**
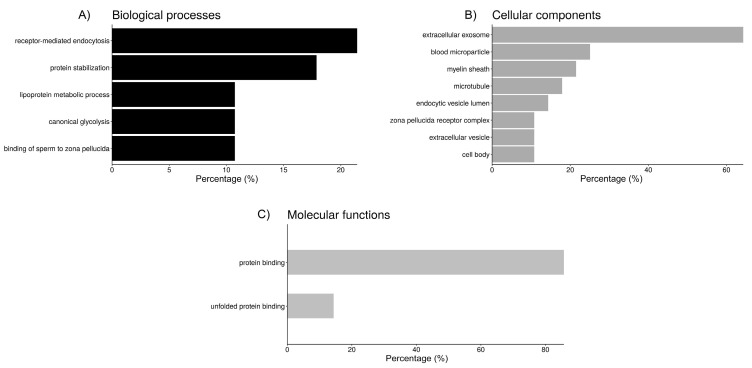
Bar chart representing the distribution of the proteins identified in spermatozoa incubated with uterine fluid (UF) according to the biological process (**A**), cellular component (**B**), and molecular function (**C**).

**Figure 3:**
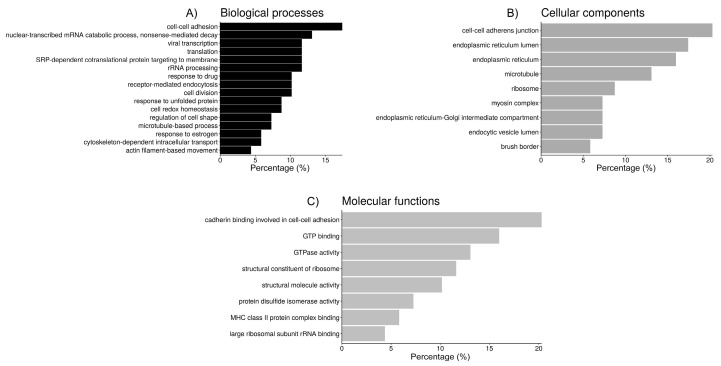
Bar chart representing the distribution of the proteins identified in spermatozoa incubated with oviductal fluid (OF) according to the biological process (**A**), cellular component (**B**), and molecular function (**C**).

**Figure 4:**
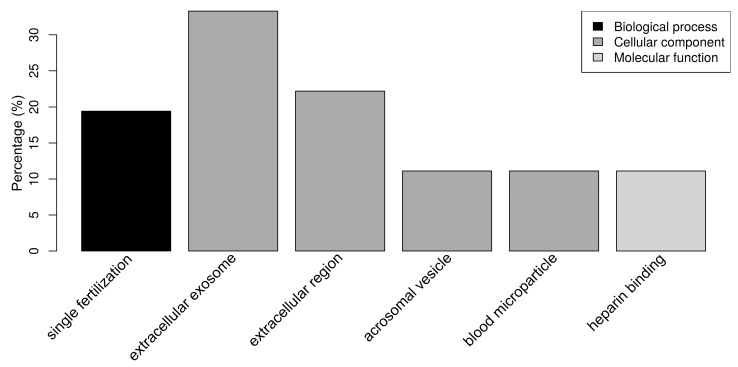
Bar chart representing the distribution of the proteins identified in spermatozoa incubated with seminal plasma (SP) according to the biological process, cellular component, and molecular function.

Functional annotation of the proteins from each fluid interacting with sperm was performed using DAVID (Figure 2, Figure 3 and Figure 4). Regarding the sperm groups containing UF (Figure 2), according to GO analysis, there are five relevant biological processes. Six out of the 27 proteins of the UF samples are involved in “receptor-mediated endocytosis” (21.4%), another 5 participate in “protein stabilization” (17.9%), 3 are enzymes (10.7%) involved in “canonical glycolysis”, and other 3 participate in the “binding of sperm to zona pellucida”, while the remaining significant hit includes 2 apolipoproteins and albumin, involved in the “lipoprotein metabolic process” (Figure 2). Cellular component category classification of UF proteins was enriched in the terms “extracellular exosome” (64.3%), “blood microparticle” (25%), “myelin sheath” (21.4%), “microtubule” (17.9%), and “endocytic vesicle lumen” (14.3%), “zona pellucida receptor complex” (10.7%), “extracellular vesicle” (10.7%), and “cell body” (10.7%) (Figure 2). Moreover, “protein binding” (85.7%) and “unfolded protein binding” (14.3%) were the two molecular functions that were statistically meaningful within the UF proteins interacting with the spermatozoa (Figure 2).

Clustering of the biological annotations for UF proteins showed limited results. The first cluster (Table 3), with an enrichment score (ES) of 3.4, shows proteins present in blood microparticles, some involved in receptor-mediated endocytosis, and the lipoprotein metabolic process. The second cluster (ES = 2.8) includes proteins involved in protein stabilization, which can bind unfolded proteins. Some of these proteins are allocated in the zona pellucida receptor complex.

In the OF proteins, according to the GO analysis, there were 90 different annotations with an FDR < 5%, so clustering to group the most relevant categories could be performed in order to extract more meaningful information out of the data (Table 4 and Figure 3). The first cluster (ES = 9.2), includes 14 proteins (20.3%) with the molecular function “cadherin binding involved in cell-cell adhesion”, all of them located within the “cell-cell adherens junction”, and 12 (17.4%) involved in the process of “cell-cell adhesion”. The second cluster (ES = 5.2) groups 14 proteins (20.3%), most of them (13) located within the endoplasmic reticulum (taking into account the terms “endoplasmic reticulum” and “endoplasmic reticulum lumen”, which share some redundancy, 5 of those proteins are also located in the “endoplasmic reticulum-Golgi intermediate compartment”), 6 of them (8.7%) participate in maintaining cell redox homeostasis, and 5 of them (7.2%) have protein disulfide isomerase activity. The third cluster (ES = 4.2) includes mainly ribosomal proteins, where six proteins (8.7%) are assigned to the more general location “ribosome” while other five are located in the “cytosolic large ribosomal subunit” (7.3%). Their molecular functions include “structural constituent of ribosome” (8 proteins, 11.6%) and “large ribosomal subunit rRNA binding” (3 proteins, 4.3%). These proteins participate in several biological processes, such as “nuclear-transcribed mRNA catabolic process, nonsense-mediated decay” (13%), “SRP-dependent cotranslational protein targeting to membrane” (11.6%), “viral transcription” (11.6%), “translational initiation” (11.6%), and rRNA processing (11.6%), which are all involved in the more general process “translation” (11.6%). The fourth cluster (ES = 4.1) groups seven proteins (10.1%) related to “receptor-mediated endocytosis”, and because of that five of them (7.2%) are located in the “endocytic vesicle lumen”. The fifth cluster (ES = 4.0) includes six proteins (8.7%, most of them chaperones) involved in “response to unfolded proteins”, and four proteins (5.8%) that can bind the MHC-II complex. The sixth cluster (ES = 3.8) groups different tubulins with a few related proteins (for a total of 17 proteins or a 24.6%), and the cellular location of nine of these components is the microtubule (13.0%). Eleven of these proteins can bind GTP (16%), nine have “GTPase activity” (13.0%), and five are involved in microtubule-based processes, such as “cytoskeleton-dependent intracellular transport” (4 proteins, 5.8%) or “cell division” (7 proteins, 10.1%). The “structural molecule activity” of seven of those proteins is due to their role as a “structural component of the cytoskeleton”. The seventh cluster (ES = 3.2) includes five myosins (7.2%), which are located in myosin complexes (three of them—MYH14, MYH9, MYH10—within the “myoxin II complex”, redundant with the terms “myosin II filament” and “actomyosin”, all of them participating in “actin filament-based movement” and with “microfilament motor activity”) and can bind actin filaments together with CFL1. Four are located to the “brush border” and have “actin-dependent ATPase activity” and “motor activity”. Those myosins, plus annexin ANXA1 and solute carrier channel SLC9A3R1, are involved in the “regulation of cell shape”. Finally, the eighth cluster (ES = 2.3) groups seven (10.1%) proteins participating in “response to drugs”, in which four (5.8%) are involved in the “response to estrogen”, including two heat-shock proteins and two apolipoproteins.

Regarding the SP proteins, “single fertilization” (17.1%) was the only relevant biological process (Figure 4). The SP proteins were classified as belonging to the cellular components “extracellular exosome” (34.3%), “extracellular region” (20.0%), “acrosomal vesicle” (11.4%), and “blood microparticle” (11.4%) (Figure 4). “Heparin binding” (11.1%) was the only relevant molecular function, due to the presence of the spermadhesins AWN, BSP1, AQN-1, and SPMI. It should be noted that 29 out of 42 SP proteins (69.0%) had at least one annotation related to cellular components, while 26 out of 29 (61.9%) had at least one annotation related to molecular functions, which is in contrast with the OF and UF datasets that are composed of better known proteins.

**Figure 5:**
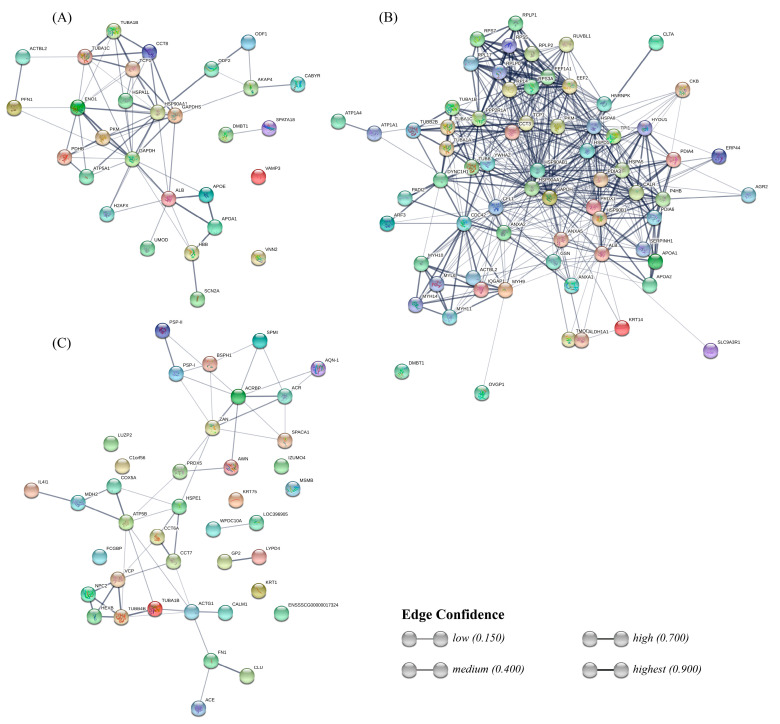
STRING network showing the interactions between uterine fluid (UF) sperm-interacting proteins (**A**), oviductal fluid (OF) sperm-interacting proteins (**B**), and seminal plasma (SP) sperm-interacting proteins (**C**).

The interactions between sperm-interacting proteins from each fluid were predicted with the STRING database (Figure 5). Regarding the UF group, this analysis revealed a strong interaction between blood plasma proteins ALB, APOE, APOA1, and HBB, and the sodium voltage channel SCN2A. The rest of the graph connects glycolysis-involved, proteins such as PDHB, PKM, ENO1, and GAPDHS, with heat shock proteins HSP90AA1 and HSPA1L, and with chaperone complex proteins TCP1 and CCT8, which in turn interact with tubulins TUBA1C and TUBA1B. HSP90AA1 has a strong bond with the outer dense fiber protein ODF2, which is intimately related to ODF1.

Interactions of proteins from the OF group form a dense net connecting several kinds of proteins. On top, ribosomal proteins form a clustering, which interacts both with tubulins and endoplasmic reticulum proteins, including several heat shock proteins and protein-disulfide isomerases. Those endoplasmic reticulum proteins interact in turn with plasma proteins ALB and apolipoproteins. In the bottom-left side, a cluster includes myosins interacting with other proteins, such as IQGAP1, CCDC42, and CFL1, which connect this cluster with the others mentioned previously. Annexins (ANXA1, 2, and 5) present bonds between each other and also interact with CCDC4, CFL1, DYNC1H1 (from the tubulins cluster), and GAPDHS. The most characteristic protein from the OF, OVGP1, only shows a weak bond to MYH9.

Interactions between SP proteins include less strong bonds between each component compared to the UF and OF protein interaction networks. One of the clusters connects boar spermadhesins (PSP-I, PSP-II, AWN, AQN-1, BSPH1) with acrosomal and sperm head proteins, such as ACR, ACRBP, ZAN, and SPACA1. The other cluster includes interactions between tubulins and NPC2, VCP, and HEXB, also bonding with chaperonin TCP1 complex proteins CCT7, CCT8A, and HSPE1, and with ACTG1 and CALM1. Both are connected with a mini-cluster involving IL4I1, MDH2, COX5A, and ATP5B. Two strong interactions remain in the network: FN1-CLU and GP2-LYPD4.

## 3. Discussion

During the journey of sperm from ejaculation to the oocyte, proteins from reproductive fluids participate in biological events that precede fertilization. For this reason, in our study, the experimental groups were designed to allow us to observe changes in the sperm proteome induced by the contact with different biological fluids. Proteins interacting with sperm cells were analyzed after the interaction with female reproductive fluids (UF and OF) in the presence or absence of SP. In order to provide new insights into the boar ejaculated spermatozoa proteome, a comparative proteome analysis of spermatozoa samples by HPLC-MS/MS was performed. The application of this technology allowed us to detect a total of 265 proteins, but when considering proteins that were present in at least 60% of the total replicates, the final number of proteins was reduced to 122. Our results showed that changes in the sperm proteome depend on the interaction with the surrounding reproductive fluids. In general, when spermatozoa were co-incubated with two fluids (SP + UF or SP + OF), a masking effect may occur towards proteins detected in single-fluid incubation. Additionally, a lower number of sperm-interacting proteins were detected from UF with respect to OF incubation, suggesting a greater adhesion of OF proteins than UF proteins to spermatozoa.

### 3.1. Uterine Fluid (UF)-Sperm Protein Interaction

The number of SP + UF sperm-interacting proteins was lower when compared to spermatozoa incubated with SP or UF. The SP + UF group showed 19 proteins in common with SP and UF groups, and 2 additional proteins (apolipoprotein A-1, deleted in malignant brain tumors 1 protein) in common with UF. Focusing on 25 exclusive sperm-interacting proteins from UF, to the best of the authors’ knowledge, few of these have been previously identified in boar and might play a role during fertilization. Regarding apolipoprotein-E, it was found in blood [46] and expressed in human endometrium during the proliferative phase [47,48,49]. Within the uterus, this protein is involved in cholesterol uptake, immune response, and preparing the endometrium for embryo implantation [50]. Two of the exclusive proteins from the UF group, vanin 2 and uromodulin, participate in the inflammatory response. Vanin 2, present in several tissues (i.e., lung, colon, placenta) and mostly in leukocytes, in soluble form or associated to the membrane, has been described in humans with a role in leukocyte migration towards inflammatory sites [51,52,53,54]. Moreover, this protein has been shown in mare and ewe UF [32,55], and in sows’ endometrium during the proliferative phase [49]. Uromudolin, which has not been previously described in UF but is a glycoprotein identified in the surface epithelium of the oviduct and uterus in mice, is involved in inhibiting the immune response [56]. It is known that, after spermatozoa deposition, an influx of PMN granulocytes occurs within the uterus, contributing to sperm selection [6,57]. Therefore, the binding of vanin 2 and uromodulin to the sperm surface and their role in the immune response could be crucial to eliminating abnormal sperm cells within the uterus. Another protein, belonging to the family of heat shock proteins, the heat shock 70-kDa protein 1-like, was detected in spermatozoa incubated with UF. This protein participates in several physiological processes (i.e., cell cycle, cell proliferation, and differentiation) and has been identified in UF from different species, such as human [47,58], equine [55], ovine [32], mouse [59], bovine, and porcine [60]. In particular, the binding of this protein to porcine and bovine spermatozoa during their journey from the uterus towards the oviduct avoids early capacitation and aids in the maintenance of sperm viability [59,61,62]. To support our findings, the study of the transcriptome of sows’ endometrium showed the presence of heat shock 70-kDa protein 1-like during the same stage of the estrus cycle of our study [49]. Among UF sperm-interacting proteins, profilin was detected on sperm for the first time to the best of our knowledge. This protein has been identified in sows’ endometrium during the late follicular phase [49] and it is known to be involved in embryo implantation [63].

Interestingly, the incubation of sperm with UF showed a higher number of detected proteins than the incubation with SP plus UF. One hypothesis could be that the combination of SP and UF leads to several interactions between each other’s proteins, resulting in less adhesion of UF proteins to the sperm. The effect of these proteins over the sperm is something worth pursuing and investigating.

After incubation with SP, UF, or SP + UF, spermatozoa showed common proteins, such as clusterin and chromosome 1 open reading frame 56. Regarding the latter, a high level of mRNA expression in the corpus and cauda epididymis, where spermatozoa acquire motility and fertilizing ability, was shown in boar [64]. The role of clusterin in sperm fertility was identified in the inner plasmatic membrane of mouse and human sperm [65,66]. Our proteomic analysis is consistent with the findings from a previous study, where clusterin was identified in human SP, decreasing DNA fragmentation and improving sperm morphology [67]. Moreover, the expression of clusterin has been previously shown in human, horse, and sheep UF [32,47,55], and in the endometrial transcriptome during the proliferative phase in pigs [48,49], supporting our findings.

When spermatozoa were incubated with UF or SP, some common proteins were detected, such as sperm equatorial segment protein 1 precursor. This protein has been described in previous studies localized in the sperm equatorial segment, and it is said to be necessary for acrosome reaction, after which it disappears, as shown in mice [68,69].

### 3.2. Oviductal Fluid (OF)–Sperm Protein Interaction

In the case of the OF sperm-interacting proteins, when spermatozoa were incubated with OF or SP plus OF, a higher number of proteins were detected in respect to the SP group. Spermatozoa incubated with OF or SP + OF showed 35 proteins in common. Among these, annexin 1, 2, and 5 were detected. The annexin 2 was described by Teijeiro et al. [70], showing its role in sperm–oviduct interaction: This protein, together with annexins 1 and 5, was identified in bovine oviduct epithelium and OF exosomes, in which annexins 1 and 2 are the most abundant, contributing to form the sperm reservoir within the oviduct [71,72,73,74]. In fact, it has been shown the interaction between the bovine SP proteins, expressed on the sperm membrane, and the annexins of the oviductal epithelium [74]. Therefore, annexins identified in the OF could mediate this interaction, as suggested by Mondejar et al. [75]. Annexin 1 is also known as an immunoregulatory protein, modulating the immune response mediated by leukocytes (reviewed by Sheikh and Solito [76]). Thus, annexin 1 could play a key role during the pre-ovulatory stage, when an increase of PMN granulocytes occurs in the oviductal environment, protecting the oviduct from the presence of any pathogenic microorganisms, as shown in bovine [77]. In our study, the detection of annexins 1, 2, and 5 on the boar sperm membrane corroborates their recruitment from OF, since our OF was from the pre-ovulatory stage of the estrus cycle. Additionally, ribosomal proteins (60S acidic ribosome protein P1, 60S acidic ribosome protein P2, 60S ribosome protein L4) were detected in sperm incubated with OF or SP plus OF. The results provided are in accordance with a previous study in which these proteins were identified in oviduct exosomes in the bovine species, showing the abundance of mRNA encoding ribosomal proteins, including 60S among others, although their role is not yet fully known [72]. Gelsolin is another protein detected in the same experimental groups, known to be characteristic of the oviductal exosomes, and involved in sperm–oviduct interaction (reviewed by Almiñana and Bauersachs [78]). This protein was described for the first time as an OF protein that binds to spermatozoa by Elliot et al. [62]. Additionally, gelsolin has been identified in OF from bovine [72], rabbit [31], human, and sheep [32]. This protein was also detected on sperm, and its localization changes according to the status of capacitation. In the first stage, it is localized in the post-acrosomal region and flagellum, while during capacitation, it moves to the acrosome region [79]. Our data also showed the presence of oviductin, which is also in accord to previous studies where oviductin recruitment on the sperm membrane has been shown [62]. This protein is known to be present in OF from different species [38,80], such as pig, bovine, hamster, and human, where its binding to the head sperm plasma membrane was shown to have an important role inducing sperm capacitation [81,82]. Moreover, oviductin is involved in tyrosine phosphorylation of sperm proteins during in vitro capacitation, confirming its role in sperm fertility [82,83].

Some of the proteins identified in spermatozoa incubated with OF or SP + OF belong to the heat shock proteins family, such as endoplasmin, heat shock cognate 71 kDa protein, 78 kDa glucose-regulated protein, and heat shock protein HSP 90-beta. These proteins have been previously identified in porcine OF [84]. Regarding the heat shock protein HSP 90-beta, belonging to the 90 kDa heat shock proteins family, it is known to bind to the sperm surface during sperm maturation within the epididymis [85]. The HSP 90-beta is also required for the maintenance of sperm motility. This fact has been corroborated by the inhibition of the heat shock 90-beta by using an HSP inhibitor (geldanamycin), resulting in a decrease in sperm motility and increase in apoptosis [86,87]. Elliot et al. [62] identified the HSP 90-beta in boar OF, demonstrating its binding to the sperm surface, supporting our findings. Furthermore, spermatozoa incubated with OF or SP + OF showed the protein disulfide-isomerase PDIA4, known to play a role in gamete interaction in mammals [88,89]. In cattle, this protein may be associated with the endoplasmin, forming a complex involved in zona pellucida hardening [84]. The presence of this protein was also shown on the mouse sperm head, where it participates in sperm–zona pellucida interaction, and it is enrolled in sperm capacitation [90]. IQGAP1 is another protein of interest that was detected in our OF and SP + OF experimental groups but also in oviductal exosomes. This protein is expressed in oviductal exosomes through the whole estrous cycle, as shown in bovine [72], but to the best of our knowledge, there were no evidence of its presence in pigs until now. Spermatozoa incubated with OF or SP + OF also showed the myosin heavy chain 9, a protein previously described in human spermatozoa, where it plays a role in oviductin binding to the sperm surface [91]. Lamy et al. [92] suggested the formation of a complex between myosin heavy chain 9 and two other proteins, oviductin and actin cytoplasmic 1, involved in modulating the capacitation process. Another protein, cofilin-1, was detected in the OF and SP + OF groups, and it is a protein that participates in sperm capacitation, inducing the acrosome reaction, as shown in humans [93]. To support our findings, cofilin-1 has been previously identified in the oviductal transcriptome in pigs [49] and in the prostasomes from human, equine, and bovine SP [94], suggesting its recruitment from both fluids. When spermatozoa were incubated with OF, some exclusive proteins were identified, whose role related to fertility is still unknown. Among these proteins, it is worth mentioning 40S ribosomal protein S3a, 40S ribosomal protein S5, peptydil arginine deiminase 2, peroxiredoxin 1, and RuvB-like helicase. The genes of these proteins have been previously described in sows’ oviductal transcriptome [49], confirming our findings in the OF group. Even when spermatozoa were incubated with SP plus OF, exclusive proteins were shown, such as anterior gradient 2 protein disulphide isomerase, keratin 14, heterogeneous nuclear ribonucleoprotein K isoform X1, Na(+)/H(+) exchange regulatory cofactor NHE-RF, and serine/threonine-protein phosphatase 2A 65 kDa regulatory subunit A alpha isoform, previously identified in the oviductal transcriptome in pig [49], thus supporting our data.

### 3.3. Seminal Plasma (SP)-Sperm Protein Interaction

Spermatozoa incubated with SP showed a lower number of proteins detected in comparison to the incubation with UF and SP + UF, whereas a higher number of SP proteins were identified in comparison to the OF and SP + OF groups. On the one hand, the higher amount of proteins from the SP + UF group with respect to SP proteins would show major protein adhesion to the sperm surface in the presence of both reproductive fluids. On the other hand, the incubation with OF or SP plus OF would show a possible masking effect from these fluids towards SP sperm-interacting proteins. Particularly, 10 exclusive proteins were detected as SP sperm-interacting proteins in comparison with UF and SP + UF groups, whereas 26 proteins in comparison with OF and SP + OF.

Spermatozoa incubated with UF or SP plus OF showed T-complex protein 1 subunit alpha, a chaperonin whose role is folding actin and tubulin [95]. It is a sperm protein involved in sperm-zona pellucida interaction in mice and humans [96,97,98], previously identified in human, equine, and bovine SP [94]. Additionally, the gene of this protein is present in sows’ endometrial and oviductal transcriptome during the late follicular phase [49]. ATP5F1B protein was detected in spermatozoa incubated with UF, co-incubated with SP plus UF, and incubated with OF. This protein is involved in maintaining sperm metabolism, necessary for sperm functions’ support, which requires ATP mainly produced by two pathways, glycolysis and mitochondrial oxidative phosphorylation [99,100]. This protein was not detected in sperm incubated with OF plus SP, suggesting a masking effect by this co-incubation on ATP5F1B protein.

### 3.4. Common Sperm Proteins after Interaction with Different Reproductive Fluids

Spermatozoa incubated with UF, OF, or SP + OF showed the heat shock protein HSP 90-alpha, previously identified among OF proteins that bind to boar and bull spermatozoa [62], and in sow endometrium during the proliferative phase [49]. This protein is known to be involved in sperm motility and maturation [101], and it has been identified in oviduct exosomes, participating in sperm–oviduct interaction in bovine species [72]. Heat shock protein HSP 90-alpha is also known to play a role in sperm capacitation, undergoing tyrosine phosphorylation during this process [102].

In spermatozoa incubated with UF, SP + UF, or SP + OF, apolipoprotein A-1 was detected. This protein was previously identified in pig SP [103] and the bovine oviduct [104], where it induces a loss of cholesterol from the sperm plasma membrane. It is known that the loss of cholesterol is a key factor regulating sperm motility, hyperactivation, and capacitation, due to the association between apolipoprotein A-1 and high-density lipoprotein, triggering the acrosome reaction [105,106]. Subsequently, apolipoprotein A-1 was identified in the UF during the secretory phase in human [47], horse [55], and more abundant in the estrus than the luteal phase in sheep [32]. In sows, this protein has also been identified in the endometrium transcriptome at day 14 of the estrus cycle [48].

When spermatozoa were incubated with SP, UF, OF, or SP + OF, calmodulin was identified. This protein is known to act as an intermediate protein of capacitation [107,108], by binding calcium after an increase of bicarbonate and calcium [109]. The presence of calcium-binding proteins could limit the free calcium, preventing early capacitation [110]. This protein, known to be expressed in spermatozoa, could also be present in SP or UF exosomes. In fact, it has been identified in human SP extracellular vesicles [111] and in UF from humans, horses, and sheep [32,47,55]. A recent analysis of the transcriptome in porcine endometrial and oviductal tissue showed the presence of calmodulin at day 18 of the estrus cycle (corresponding to the late follicular phase) [49], supporting the results of the current study.

Focusing on common proteins detected in UF, SP + UF, OF, and SP + OF, DMBT1 can be mentioned. This protein was shown to be present in the cervical mucus of sheep and Rhesus monkey in the luteal phase, when progesterone levels increase, suggesting a correlation between DMBT1 production and progesterone levels within the endometrium [44,112]. In our study, late follicular phase fluids were used, demonstrating the presence of this protein in the pig during this stage of the cycle for the first time. To support this finding, an endometrial transcriptome analysis in pigs revealed the presence of DMBT1 mRNA during the late follicular phase [49]. DMBT1 expression had already been shown in oviductal epithelium and oviductal fluid from porcine [75,113,114,115], bovine [116], and human [117].

All groups except spermatozoa incubated with OF showed two proteins belonging to spermadhesins, PSP-I and PSP-II. It is interesting to note that the OF group did not show these proteins, since all spermatozoa used for the experiment were in contact with SP during the ejaculation. In fact, these proteins belong to SP [118,119] that adhere to the sperm membrane, regulating motility and fertilizing ability [11]. In particular, PSP-I is involved in preventing an early acrosome reaction [120]. Likewise, PSP-I forms a heterodimeric complex with PSP-II, involved in the immune response within the uterus after semen deposition [121]. This complex, together with AQN-1 (detected in all experimental groups from our study), bound to the membrane, playing a protective role towards the sperm membrane, viability, motility, and mitochondrial activity [122]. The sperm adhesin AWN was detected in all experimental groups, as expected. This protein is known to bind to the sperm surface during ejaculation and leads spermatozoa during their transit within the female reproductive tract up to the zona pellucida, avoiding early capacitation, a process that occurs when AWN is removed from the sperm surface (reviewed by Rodriguez-Martinez et al. [26]). Kim et al. [49] showed that AWN mRNA has not been detected in sows’ endometrium and oviduct during the proliferative phase. Thus, the AWN detected in our work may have a male origin as previously described [123].

## 4. Material and Methods

### 4.1. Ethics

All the procedures carried out in this work were approved on 31 July 2019 (PGC2018-094781-B-100) and on 1 June 2020 (PID2019-106380RB-I00) by the Ethical Committee of Animal Experimentation of the University of Murciaand.

### 4.2. Ejaculated Spermatozoa Collection

Ejaculated sperm samples were collected by manual technique from 5 boars with proven fertility (CEFU S.A., Murcia, Spain). All boars were kept in abstinence during 3–4 days before ejaculate collection. Quality criteria were applied to each sample before use (rich fraction volume ≥ 75 mL, concentration ≥ 200 × 10^6^ sperm/mL, motility ≥ 70%, and viability ≥ 85%). Spermatozoa concentration was calculated by SpermaCue photometer (Minitub, Germany).

### 4.3. Collection and Preparation of Biological Fluids (SP, UF, and OF)

The SP was obtained by ejaculate centrifugation at 13,800× *g* (Model 5418 R, Eppendorf^®^, Hamburg, Germany) for 10 min at 4 °C. Then, the supernatant was collected and centrifuged at 13,800× *g* for further 10 min at 4 °C to remove cell debris and any remaining spermatozoa (microscopically verified). The SP samples were then separated into aliquots and stored at −80 °C (New Brunswick Premium u570 ULT Freezer) until use. Boar SP from 3 different males was mixed in a single pool to perform all the experiments.

The UF and OF were collected from genital tracts obtained at the slaughterhouse (El Pozo S.A., Alhama de Murcia, Murcia, Spain). The female tracts were selected based on the appearance of the ovary (preovulatory stage (follicles of 8–11 mm Ø)) [124]. The female genital tracts were transported to the laboratory within 60 min after collection. The UF was extracted through a mechanical pressure from the uterine tubal junction to the end of the horns, followed by double centrifugation at 7200× *g* for 10 min at 4 °C to remove debris, and stored in aliquots at −80 °C until use. In order to obtain OF, the oviducts were separated from the genital tracts and quickly washed with 0.4% *v/v* cetrimide (alkyltrimethylammonium bromide) (Sigma-Aldrich, Darmstadt, Germany) solution and twice in phosphate buffer solution without calcium and magnesium (PBS; Sigma-Aldrich^®^, Madrid, Spain) and transferred to Petri dishes on ice to be dissected. After the dissection, the OF was collected by aspiration with an automatic pipette by introducing the tip into the ampulla, applying a pressure from the isthmus to the ampulla as described previously [124]. The OF was centrifuged twice at 7200× *g* for 10 min at 4 °C to remove debris. The supernatant was stored in aliquots at −80 °C until use. A pool of each fluid (UF and OF) from at least 3 different females was used for all the experiments.

### 4.4. Spermatozoa Incubation with Biological Fluids

Ejaculated spermatozoa were centrifuged at 500× *g* for 10 min to eliminate the SP and diluted in PBS. The spermatozoa concentration was calculated by a SpermaCue photometer to reach a concentration of 20 × 10^6^ sperm/mL and six different groups were prepared: (1) SP group: spermatozoa with 20% of SP in PBS; (2) UF group: spermatozoa with 20% of UF in PBS; (3) OF group: spermatozoa with 20% of OF in PBS; (4) SP + UF group: spermatozoa with 20% of UF and 20% of SP in PBS; (5) SP + OF group: spermatozoa with 20% of OF and 20% of SP in PBS. The concentration of the different fluids used was based on previous studies performed in the pig [125] and other species [126,127,128]. Moreover, preliminary experiment tests were performed and indicated that a 20% or higher concentration has no evident impact on the sperm physiology (unpublished data).

All the groups were incubated for 180 min at 38 °C. After the incubation period, samples were centrifuged at 600× *g* for 5 min to eliminate the supernatant and pellet was stored at −20 °C until use for protein extraction.

### 4.5. Protein Extraction

The semen samples (15 × 10^6^ sperm/mL) were washed twice in PBS held at 4 °C (500 µL) and centrifuged at 900× *g* for 15 min at 4 °C. The supernatant was carefully discarded, and the pellet was resuspended in 300 µL of lysis solution (25 mM ammonium bicarbonate buffer pH 8.5 and 0.01% ProteaseMax Surfactant (Promega, Madrid, Spain)). The sonication procedure, performed by Branson Ultrasonic Sonifier SLPe (Apollo Ultrasonics, York, UK), was achieved by 5 cycles of 10 s of sonication (40% amplitude) and 30 s of ice incubation. The cell lysate was centrifuged at 15,000× *g* for 15 min at 4 °C. The supernatant was transferred to a fresh microfuge tube to determine the protein concentration by Bradford assay. For proteomics analysis, 100 µg of protein were used.

### 4.6. Trypsin Digestion

Each sample was dissolved in 100 µL of 50 mM ammonium bicarbonate (Sigma-Aldrich, Madrid, Spain) buffer pH 8.5 with 0.01% ProteaseMax (Promega, Madrid, Spain), which enhances the trypsin digestion. Protein samples were reduced by adding 20 mM DTT (1,4-dithiothreitol; Sigma-Aldrich, Madrid, Spain) at 56 °C for 20 min. Then, samples were alkylated by adding 100 mM IAA (indole-3-acetic acid sodium salt; Sigma-Aldrich, Madrid, Spain) during 30 min at room temperature in the dark. Finally, digestion by was performed adding 1 µg of Trypsin Gold Proteomics Grade (Promega, Madrid, Spain) (approx. 1:100 *w*/*w*) during 3 h at 37 °C. Reaction was stopped with 0.1% formic acid (Fisher Scientific, Madrid, Spain) and filtered through a 0.2-µm filter (Pall Corporation, Madrid, Spain). After that, samples were dried using an Eppendorf Vacuum Concentrator model 5301 (Sigma-Aldrich, Madrid, Spain).

### 4.7. High-Performance Liquid Chromatography-Mass Spectrometry Analysis (HPLC-MS/MS Analysis)

The separation and analysis of the tryptic digestion of the samples were performed with a high-performance liquid chromatography-mass spectrometry (HPLC/MS) system consisting of an Agilent 1290 Infinity II Series HPLC (Agilent Technologies, Santa Clara, CA, USA) equipped with an Automated Multisampler module and a High-Speed Binary Pump, and connected to an Agilent 6550 Q-TOF Mass Spectrometer (Agilent Technologies, Santa Clara, CA, USA) using an Agilent Jet Stream Dual electrospray (AJS-Dual ESI) interface. Experimental parameters for HPLC and Q-TOF were set in MassHunter Workstation Data Acquisition software (Rev. B.08.00, Agilent Technologies, Santa Clara, CA, USA).

Dry samples from trypsin digestion were resuspended in 20 µL of buffer A, consisting of water/acetonitrile (Fisher Scientific)/formic acid (94.9:5:0.1). Sample was injected onto an Agilent AdvanceBio Peptide Mapping HPLC column (2.7 µm, 100 × 2.1 mm, Agilent technologies, Santa Clara, CA, USA), thermostatted at 55 °C, at a flow rate of 0.4 mL/min. This column is suitable for peptide separation and analysis. After the injection, the column was washed with buffer A for 2 min and the digested peptides were eluted using a linear gradient 0–40% B (buffer B: water/acetonitrile/formic acid, 10:89.9:0.1) for 30 min.

The mass spectrometer was operated in the positive mode. The nebulizer gas pressure was set to 35 psi, whereas the drying gas flow was set to 14 L/min at a temperature of 300 °C, and the seath gas flow was set to 11 L/min at a temperature of 250 °C. The capillary spray, fragmentor, and octopole RF Vpp voltages were 3500, 360, and 750 V, respectively. Profile data were acquired for both MS and MS/MS scans in extended dynamic range mode. The MS and MS/MS mass range was 50–1700 m/z and scan rates were 8 spectra/s for MS and 3 spectra/s for MS/MS. Auto MS/MS mode was used with precursor selection by abundance and a maximum of 20 precursors selected per cycle. A ramped collision energy was used with a slope of 3.6 and an offset of −4.8. The same ion was rejected after two consecutive scans.

Data processing and analysis was performed by Spectrum Mill MS Proteomics Workbench (Rev B.06.00.201, Agilent Technologies, Santa Clara, CA, USA). Briefly, raw data were extracted under default conditions as follows: Unmodified or carbamidomethylated cysteines; [MH] + 50–10000 m/z; maximum precursor charge + 5; minimum signal-to-noise MS (S/N) 25; finding ^12^C signals.

The MS/MS search against the appropriate and updated protein database was performed with the following criteria: Variable modifications search mode (carbamidomethylated cysteines, STY phosphorylation, oxidized methionine, and N-terminal glutamine conversion to pyroglutamic acid); tryptic digestion with 5 maximum missed cleavages; ESI-Q-TOF instrument; minimum matched peak intensity 50%; maximum ambiguous precursor charge + 5; monoisotopic masses; peptide precursor mass tolerance 20 ppm; product ion mass tolerance 50 ppm; and calculation of reversed database scores. Validation of the peptide and protein data was performed using auto thresholds.

### 4.8. Bioinformatic Analysis

#### 4.8.1. Venn Diagram

A Venn diagram comparing the five experimental groups was generated with library “venn” (Dusa 2018) in R 3.5.1 (R Core Team 2018) [129].

#### 4.8.2. Annotation of Human Homologs and Gene Ontology Analysis

Raw Uniprot IDs from the proteomics results Excel^®^ file were used to look for the most completely annotated IDs in *Sus scrofa* and its corresponding homologs from human species querying the UniProt API (UniProt Consortium, 2018) [130] with a custom script written in Python 2.7. Uniprot IDs were then annotated with the functional annotation tool of the Database for Annotation, Visualization and Integrated Discovery (DAVID, version 6.8) [131]. Three independent sets of ontology were used: “molecular function”, “biological processes”, and “cellular components”. Most statistically significant (FDR < 5%) GO terms were checked with REVIGO [132] to discard redundant terms. The percentages of selected GO terms were then plotted with a custom R script making use of libraries dplyr [133] and ggplot2 [134].

Moreover, a classification of the proteins present in each fluid was made throughout the functional clustering tool of DAVID.

#### 4.8.3. Protein-Protein Interaction Network

Lists of proteins corresponding to sperm-interacting UF and OF proteins were uploaded to STRING [135] and mapped to the human species database, which includes more interactions than the porcine database. The SP proteins were mapped to the porcine database due to the existence of many exclusive proteins [120] not present in the human proteome. The OF proteins’ net of interactions was clustered using the MCL algorithm [136] with the inflation parameter set to 3.

## 5. Conclusions

In conclusion, our study provided novel knowledge regarding proteins that adhere to boar ejaculate sperm’s surface after contact with male and female reproductive fluids. The present work highlights how the sperm proteome changes during their journey within the female reproductive tract. The combined use of SP with UF or OF suggests an interaction between these fluids that modifies the sperm proteins, most likely caused by a steric hindrance. Thus, changes in the sperm proteome may have a potential physiological impact during the in vivo fertilizing process.

## Figures and Tables

**Table 1:** List of boar spermatozoa proteins identified after incubation with uterine fluid (UF group), seminal plasma (SP group), and uterine fluid and seminal plasma (SP + UF group).

Experimental Groups	Protein Name	Accession Number
UF	A-kinase anchoring protein 4	A0A286ZWH7
	Apolipoprotein E	P18650
	ATP synthase subunit alpha	A0A287BBS4
	Chaperonin containing TCP1 subunit 8	I3LCA2
	Enolase 1	A0A287B6S5
	Glyceraldehyde-3-phosphate dehydrogenase	F1RM74
	Heat shock 70 kDa protein 1-like	A5A8V7
	Heat shock protein HSP 90-alpha	A0A287AQK7
	Hemoglobin subunit alpha	P01965
	Hemoglobin subunit beta	A0A5G2QRW3
	Histone H2A	A0A5G2QMX0
	IgA heavy chain constant region	A0A287B626
	Ig like protein	A0A286ZTC9
	Mitochondria-eating protein	A0A5G2R8N5
	Multifunctional fusion protein	A0A287B7L9
	Outer dense fiber protein 1	Q29077
	Outer dense fiber protein 2	F1RR82
	Profilin	F1RFY1
	Pyruvate dehydrogenase E1 component subunit beta	A0A5G2QSU5
	RIIa domain-containing protein	A0A5G2R5J1
	Serum albumin	A0A287BAY9
	T-complex protein 1 subunit alpha	F1SB63
	Uromodulin	F1RPA9
	Vanin 2	F1S3Q9
	V-SNARE coiled-coil homology domain-containing protein	A0A287BEC7
SP	Beta-hexosaminidase	D0G6 × 8
	Beta-microseminoprotein	A0A2C9F3B6
	Cytochrome c oxidase subunit 5A	F1SJ34
	Fc fragment of IgG binding protein	A0A287BCE6
	Glycoprotein 2	F1RPA7
	Heat shock 10kDa protein 1	F1SMZ6
	Keratin 1	F1SGG3
	NPC intracellular cholesterol transporter 2	A0A5G2RMF7
	T-complex protein 1 subunit zeta	I3L9J4
	WAP four-disulfide core domain 10A-like (WFDC10AL)	A0A287AEV7
UF *, SP *	Acrosin	A0A287AFN9
	Angiotensin-converting enzyme	F1RRW5
	Calmodulin	A0A5G2QWK6
	IZUMO family member 4	A0A5G2QPK2
	Keratin 75	F1SGI7
	LY6/PLAUR domain containing 4	D3K5J4
	Malate dehydrogenase	A0A5G2RGL7
	Peroxiredoxin 1	A0A286ZND5
	Sperm equatorial segment protein 1 precursor	A0A287B423
	T-complex protein 1 subunit eta	A0A5G2RAV7
	Transitional endoplasmic reticulum ATPase	A0A286ZUM8
	Uncharacterized protein (leucine-rich repeat-containing protein 37A-like isoform X2)	A0A287B9V6
	Zonadhesin	A0A5G2QZP6
UF *, SP + UF *	Deleted in malignant brain tumors 1 protein	Q4A3R3
	Apolipoprotein A-I	K7GM40
UF *, SP *, SP + UF *	Acrosin-binding protein	F1SL45
	Actin, cytoplasmic 1/Actin gamma 1	A0A287AA77
	Amine oxidase	F1RHU4
	ATP synthase subunit beta	K7GLT8
	Carbohydrate-binding protein AQN-1	Q4R0H3
	Chromosome 1 open reading frame 56	A0A5G2QRQ5
	Clusterin	A0A5S6I5T1
	Fibronectin 1	F1SS24
	Jacalin-type lectin domain-containing protein	A0A287AVU8
	Leucine zipper protein 2/LUZP2	A0A287BT68
	Major seminal plasma glycoprotein PSP-I	P35495
	Major seminal plasma glycoprotein PSP-II	P35496
	Seminal plasma protein pB1	A0A2C9F357
	Seminal plasma sperm motility inhibitor	I7HJH6
	Sperm acrosome membrane-associated protein 1	D5K8A9
	Spermadhesin AWN	Q4R0H8
	Sperm-associated acrosin inhibitor isoform X1	A0A2C9F3F5
	Tubulin alpha chain	F2Z5T5
	Tubulin beta chain	A0A5G2QGK1

Proteins listed without * represents exclusive proteins among the different groups, whereas the presence of * denotes common proteins between them.

**Table 2:** List of boar spermatozoa proteins identified after incubation with oviductal fluid (OF group), with seminal plasma (SP group), and with oviductal fluid and seminal plasma (SP + OF group).

Experimental Groups	Protein Name	Accession Number
OF	40S ribosomal protein S3a	F2Z5C7
	40S ribosomal protein S5	F2Z5E6
	60S acidic ribosomal protein P0	A0A5S6HGK5
	60S ribosomal protein L17 isoform a	A0A287B386
	Apolipoprotein A-II preproprotein	A0A481BCM9
	Creatine kinase B-type	A0A5G2R6 × 7
	Dynein cytoplasmic 1 heavy chain 1	A0A287B9W3
	Elongation factor 1-alpha	A0A287A391
	Glyceraldehyde-3-phosphate dehydrogenase	F1RM74
	Myosin heavy chain 14	I3LIE3
	Myosin light polypeptide 6	A0A5G2QAD4
	Oviduct-specific glycoprotein	Q28990
	Peptidyl arginine deiminase 2	I3LNE4
	Peptidylprolyl isomerase	A0A287B5T6
	RuvB-like helicase	I3L742
	T-complex protein 1 subunit gamma	A0A287AMZ2
SP	Acrosin	A0A287AFN9
	Acrosin-binding protein	F1SL45
	Amine oxidase	F1RHU4
	Angiotensin-converting enzyme	F1RRW5
	Beta-hexosaminidase	D0G6 × 8
	Beta-microseminoprotein	A0A2C9F3B6
	Chromosome 1 open reading frame 56	A0A5G2QRQ5
	Clusterin	A0A5S6I5T1
	Cytochrome c oxidase subunit 5A	F1SJ34
	Fc fragment of IgG binding protein	A0A287BCE6
	Glycoprotein 2	F1RPA7
	Heat shock 10kDa protein 1	F1SMZ6
	IZUMO family member 4	A0A5G2QPK2
	Jacalin-type lectin domain-containing protein	A0A287AVU8
	Keratin 1	F1SGG3
	Keratin 75	F1SGI7
	Leucine zipper protein 2/LUZP2	A0A287BT68
	LY6/PLAUR domain containing 4	D3K5J4
	Malate dehydrogenase	A0A5G2RGL7
	NPC intracellular cholesterol transporter 2	A0A5G2RMF7
	Seminal plasma protein pB1	A0A2C9F357
	Sperm acrosome membrane-associated protein 1	D5K8A9
	Sperm equatorial segment protein 1 precursor	A0A287B423
	Sperm-associated acrosin inhibitor isoform X1	A0A2C9F3F5
	WAP four-disulfide core domain 10A-like (WFDC10AL)	A0A287AEV7
	Zonadhesin	A0A5G2QZP6
SP + OF	40S ribosomal protein S7	A0A287A9Y6
	Anterior gradient 2, protein disulphide isomerase family member	A0A287AM82
	Apolipoprotein A-I	K7GM40
	Heterogeneous nuclear ribonucleoprotein k isoform X1	I3LQS0
	Hypoxia upregulated 1	A0A286ZZF0
	Keratin 14	F1S0J8
	Na(+)/H(+) exchange regulatory cofactor NHE-RF	B8XH67
	Serine/threonine-protein phosphatase 2A 65 kDa regulatory subunit A alpha isoform	P54612
	T-complex protein 1 subunit alpha	F1SB63
OF *, SP *	Actin, cytoplasmic 1/Actin gamma 1	A0A287AA77
	ATP synthase subunit beta	K7GLT8
	Peroxiredoxin 1	A0A286ZND5
	T-complex protein 1 subunit zeta	I3L9J4
OF *, SP+OF *	60 kDa heat shock protein, mitochondrial	A0A287ATN8
	60S acidic ribosomal protein P1	F1SIT7
	60S acidic ribosomal protein P2	A0A287B7U0
	60S ribosomal protein L4	A0A5G2QSX6
	Aldehyde dehydrogenase 1 family member A1	I3LRS5
	Annexin I	F1SJB5
	Annexin II	A0A286ZJV6
	Annexin V	F2Z5C1
	Calreticulin	P28491
	Clathrin light chain	A0A287BFJ2
	Cofilin-1	K7GK75
	Deleted in malignant brain tumors 1 protein	Q4A3R3
	Endoplasmic reticulum chaperone BiP/78 kDa glucose-regulated protein	A0A287BIL8
	Endoplasmin	Q29092
	Eukaryotic translation elongation factor 2	I3LII3
	Gelsolin	A0A287A6P1
	Heat shock cognate 71kDa protein	A0A286ZWK2
	Heat shock protein HSP 90-alpha	A0A287AQK7
	Heat shock protein HSP 90-beta	A0A286ZKC5
	IQ motif containing GTPase activating protein 1	IQGAP1
	Multifunctional fusion protein	A0A287B7L9
	Myosin 9	A0A5G2R8R0
	Myosin heavy chain 14	MYH9
	Myosin-11	MYH11
	Oviduct-specific glycoprotein	OVGP1
	Protein disulfide-isomerase P4HB	G9F6 × 8
	Protein disulfide-isomerase PDIA3	F6QA08
	Protein disulfide-isomerase PDIA4	PDIA4
	SERPIN domain-containing protein/Serpin H1 precursor	A0A286ZRU9
	Serum albumin	A0A287BAY9
	Sodium/potassium-transporting ATPase subunit alpha	F1SAX3
	Thioredoxin domain-containing protein	A0A5G2Q895
	Triosephosphate isomerase	A0A288CFT0
	Tropomodulin 3	A0A5G2R425
	Tyrosine 3-monooxygenase/tryptophan 5-monooxygenase activation protein zeta	F2Z558
OF *, SP + OF *, SP *	Calmodulin	A0A5G2QWK6
	Carbohydrate-binding protein AQN-1	Q4R0H3
	Fibronectin 1	F1SS24
	Seminal plasma sperm motility inhibitor	I7HJH6
	Spermadhesin AWN	Q4R0H8
	T-complex protein 1 subunit eta	A0A5G2RAV7
	Transitional endoplasmic reticulum ATPase	A0A286ZUM8
	Tubulin alpha chain	F2Z5T5
	Tubulin beta chain	A0A5G2QGK1
SP + OF *, SP *	Leucine-rich repeat-containing protein 37A	A0A287B9V6
	Major seminal plasma glycoprotein PSP-I	P35495
	Major seminal plasma glycoprotein PSP-II	P35496

Proteins listed without * represents exclusive proteins among the different groups, whereas the presence of * denotes common proteins between them.

**Table 3:** DAVID functional annotation clustering for sperm-interacting proteins within uterine fluid (UF).

Most Descriptive Categories of DAVID Functional Annotation Clusters by Similar GO Terms	Proteins ^1^	Score ^2^
GO Group 1: Endocytosis	9	3.4
blood microparticle (7, 30.0) ^3^		
receptor-mediated endocytosis (6, 20.1)		
endocytic vesicle lumen (4, 162.7)		
lipoprotein metabolic process (3, 49.1)		
GO Group 2: Protein folding/binding to zona pellucida	6	2.8
protein stabilization (5, 22.9)		
unfolded protein binding (4, 21.9)		
zona pellucida receptor complex (3, 217.0)		
binding of sperm to zona pellucida (3, 53.3)		
cell body (3, 31.0)		

Table shows DAVID functional annotation clusters with a score ≥ 1.3. ^1^ number of unique proteins in each GO group; ^2^ highest enrichment score (geometric mean of member’s *p*-values of the corresponding annotation cluster in −log_10_ scale) of each group; ^3^ in brackets: number of genes and fold enrichment for the functional term.

**Table 4:** DAVID functional annotation clustering for sperm-interacting proteins within oviductal fluid (OF).

Most Descriptive Categories of DAVID Functional Annotation Clusters by Similar GO Terms	Proteins ^1^	Score ^2^
GO Group 1: Cell adhesion/junction	14	9.2
cadherin binding involved in cell-cell adhesion (14, 11.8) ^3^		
cell-cell adherens junction (14, 11.4)		
cell-cell adhesion (12, 10.9)		
GO Group 2: Protein folding	14	5.2
endoplasmic reticulum lumen (12, 16.5)		
endoplasmic reticulum (11, 3.5)		
cell redox homeostasis (6, 19.2)		
protein disulfide isomerase activity (4, 56.5)		
endoplasmic reticulum-Golgi intermediate compartment (5, 19.4)		
GO Group 3: Ribosome/translation	9	4.2
nuclear-transcribed mRNA catabolic process, nonsense-mediated decay (9, 18.7)		
SRP-dependent cotranslational protein targeting to membrane (8, 21.0)		
viral transcription (8, 17.6)		
translational initiation (8, 14.4)		
rRNA processing (9.2, 8)		
structural constituent of ribosome (8, 8.8)		
translation (8, 7.8)		
ribosome (6, 9.5)		
cytosolic large ribosomal subunit (5, 19.4)		
large ribosomal subunit rRNA binding (3, 104.9)		
GO Group 4: Endocytosis	7	4.1
receptor-mediated endocytosis (7, 9.3)		
endocytic vesicle lumen (5, 82.5)		
GO Group 5:	7	4.0
response to unfolded protein (6, 35.3)		
MHC class II protein complex binding (4, 61.2)		
GO Group 6: Microtubules	17	3.8
GTP binding (11, 7.0)		
GTPase activity (9, 9.4)		
microtubule (9, 7.6)		
structural molecule activity (7, 6.9)		
cell division (7, 4.9)		
structural contstituyent of cytoskeleton (6, 13.3)		
microtubule-based process (5, 34.3)		
cytoskeleton-dependent intracellular transport (4, 54.9)		
GO Group 7: Myosin	8	3.2
actin filament binding (6, 11.0)		
myosin complex (5, 26.4)		
motor activity (5, 18.5)		
regulation of cell shape (5, 8.8)		
actin-dependent ATPase activity (4, 75.3)		
brush border (4, 17.6)		
myosin II filament (3, 264.1)		
myosin II complex (3, 113.2)		
actomyosin (3, 66.0)		
actin filament-based movement (3, 43.6)		
microfilament motor activity (3, 36.7)		
GO Group 8:	7	2.3
response to drug (7, 5.7)		
response to estrogen (4, 15.2)		

Table shows DAVID functional annotation clusters with a score ≥ 1.3. ^1^ represents number of unique proteins in each GO group; ^2^ represents highest enrichment score (geometric mean of member’s *p*-values of the corresponding annotation cluster in −log_10_ scale) of each group; ^3^ represents brackets: number of genes and fold enrichment for the functional term.

## Supplementary Materials

Supplementary materials can be found at https://www.mdpi.com/1422-0067/21/17/6060/s1. Supplementary file 1. List of proteins identified in boar ejaculated spermatozoa after incubation with seminal plasma (SP), uterine fluid (UF), seminal plasma plus uterine fluid (SP + UF), oviductal fluid (OF), seminal plasma plus oviductal fluid (SP + OF). Sperm biological functions have been listed using UniProt KB database (www.uniprot.org) in combination with PANTHER (www.panther.org). Numbers under each experimental group columns indicate the protein score and the mean number of peptides identified across the group replicates.
